# The impact of the COVID-19 pandemic on HIV healthcare delivery for females in sub-Saharan Africa: A scoping review

**DOI:** 10.1371/journal.pgph.0002975

**Published:** 2024-12-05

**Authors:** Reeya Kothari, Rachel Victoria Baran, Paulina Rudziak, Vishva Shah, Elysée Nouvet

**Affiliations:** 1 Schulich School of Medicine and Dentistry, Western University, London, Ontario, Canada; 2 Department of Health Sciences, Western University, London, Ontario, Canada; New York University Grossman School of Medicine, UNITED STATES OF AMERICA

## Abstract

Throughout sub-Saharan Africa (SSA), females are disproportionately impacted by HIV and face generalized but also gendered social and economic barriers to accessing HIV healthcare and services. At the outset of the pandemic, many anticipated COVID-19 would represent a major set-back for HIV care in the region. The impact of COVID-19 on HIV health services and HIV-affected populations has been reported in specific populations but has not been synthesized for females in the SSA region. The objective of this scoping review is to investigate the impact of COVID-19 on HIV healthcare delivery and services for females in SSA. Four databases were searched to identify relevant studies. Studies included were limited to English primary research articles reporting on the interactions between COVID-19 and HIV healthcare delivery and services amongst females in SSA. Two pairs of reviewers each screened 1390 articles via abstract and full-text screening, and data extraction was facilitated with a standardized protocol. A total of 39 studies were included in this review. Through thematic analysis of the articles, we identified five major themes—accessibility, availability, affordability, clinical barriers, and psychosocial barriers—which complicated the provision of HIV care during the COVID-19 pandemic and facilitators of HIV care during the pandemic. The literature highlighted two special populations, female sex workers and pregnant females, as having unique challenges in accessing HIV care due to societal stigma and their personalized health needs. Reviewed articles indicate that the COVID-19 pandemic introduced and exacerbated barriers to the accessibility, availability, and affordability of HIV healthcare and services for females in SSA. This review should be used by healthcare workers, healthcare administrators, policymakers, and the government to better understand the current gaps in HIV service provision to females during the COVID-19 pandemic, which can support the delivery of HIV care to females for future public health emergencies.

## Introduction

The Human Immunodeficiency Virus (HIV) global epidemic has devastated populations worldwide for over 40 years [1]. With the emergence of the COVID-19 pandemic, healthcare systems worldwide have been forced to handle the double burden of both infectious diseases. As of March 2023, over 675 million cases of COVID-19 have been reported globally [2]. The first case of COVID-19 in Sub-Saharan Africa (SSA) was reported in Nigeria on February 27^th^, 2020 [3]. Eventually, the virus spread to every country in SSA [3]. The burden of COVID-19 varies by country within SSA. South Africa reported the highest COVID-19 case count, while the highest rates of mortality were seen in both South Africa and Ethiopia [4, 5].

Each country in SSA responded to the public health emergency differently. Kenya, Uganda and Cameroon promptly initiated curfews, full lockdowns, and strict physical distancing measures. Conversely, Zambia and Tanzania did not impose curfews in the early stages of the pandemic [5]. Across SSA, countries with a higher rate of income inequality and higher median age reported higher rates of mortality [6, 7].

It was predicted that the large number of crowded informal settlements, high rate of poverty, and fragile health systems, may result in SSA facing a greater burden of COVID-19 when compared globally [4]. The COVID-19 pandemic caused widespread disruptions to all health facilities including primary care [8]. The burden of the COVID-19 pandemic resulted in the re-routing of healthcare workers, financial and physical resources from primary care to COVID-19 care. This may have had a long-lasting impact on the healthcare delivery for other infectious diseases such as HIV.

Human immunodeficiency virus is an infection that attacks the human immune system [9]. Globally, over 38 million people are living with HIV, with African regions being disproportionately affected [9]. Overall, the adult prevalence of HIV in SSA is 9.0% [10]. Currently there is no cure for HIV and if infected, an individual will have the virus for life [11]. To prevent HIV-related complications, adequate medical care is essential [9]. The main method of managing HIV is through antiretroviral therapy (ART). Antiretroviral therapy involves a combination of antiretroviral (ARV) drugs, which can increase the medication potency, reduce one’s viral load to undetectable levels, and reduce the likelihood of viral drug resistance [9, 10]. Antiretroviral drugs prevent HIV from causing further damage to the body, reduce the chance of HIV-related complications, and improve health outcomes, which is why early detection of HIV and early ART initiation are crucial [10].

National guidelines on HIV vary among countries in SSA. Countries including Nigeria, Sierra Leone and Uganda, follow the same definition for advance HIV disease as the World Health Organization (WHO) 2021 guidelines, while South Africa does not provide a definition of advance HIV disease and Malawi includes additional virological failure, hospitalization status and clinical signs in their definition [12]. All five of these countries recommend the same first line ART regiment as the WHO, however guidelines for ART in patients with tuberculosis vary [12]. This baseline variation in HIV and ART guidelines across SSA have resulted in differential impacts of HIV across the sub-continent.

Females are disproportionately impacted by HIV in SSA [13]. In SSA, the rate of HIV infection is three to seven times higher in adolescent females compared to adolescent males [13, 14]. In SSA, females face a greater burden of HIV because of various biological and social factors. Biological factors that increase a female’s susceptibility to HIV include age of sexual debut, viral load, and anatomy and epithelial integrity [13]. Since females face greater levels of poverty, gender-based violence and economic constraints, their access to healthcare is limited [13].

### Research question

The double burden of HIV and COVID-19 has placed a large burden on healthcare systems in SSA. Despite females being biologically more susceptible to HIV infection and facing many barriers to HIV care and services in this context, the potential impact of the COVID-19 pandemic on the delivery and use of these essential HIV healthcare services among females has not been reviewed in literature. The objective of this scoping review is to answer the question: what is the impact of the COVID-19 pandemic on HIV healthcare delivery and services for females in SSA? This review will identify gaps that exist in the literature and work towards developing evidence-based policies to improve HIV healthcare delivery systems for females in SSA. This is important because the evidence collected and analyzed in this literature can help inform the provision of HIV services to females during emergency public health crises in the future.

## Methods

### Study design

The international database of Prospectively Registered Systematic Reviews in health and social care (PROSPERO) was searched for similar or identical reviews prior to registration, and none were found [15]. The Preferred Reporting Items for Systematic Reviews and Meta-Analyses (PRISMA) guidelines were followed to systematically identify and assess data from included articles. The guidelines allowed for ensuring consistency among methods and analyses for this review [16].

### Inclusion and exclusion criteria

All study authors were trained on study inclusion and exclusion criteria prior to the literature selection. Inclusion criteria for titles and abstract screening consisted of primary articles in English with a study period during the COVID-19 pandemic, and related to the topics of COVID-19, HIV, female, and HIV healthcare delivery and service [17]. For the purpose of this review, the term ‘female’ encompasses individuals who were assigned the female sex at birth, people who identify as female or woman, and females of any age range. Editorials, commentaries, and reviews were excluded to focus on evidence-based primary research.

### Search strategy and selection

To inform this scoping review, the Web of Science, Medline (OVID interface), Embase (OVID interface), and Scopus databases were searched for articles from inception to 12 June, 2023. These databases were selected based on their relevance to the medical and social science field. Utilizing Covidence to review the articles allowed the reviewers to become familiarized with the data. This further enhanced the reviewers understanding of common themes and patterns that the data presented. The main key terms used for the search strategies were ‘COVID-19’, ‘HIV’, ‘female’, ‘women/woman’, and ‘sub-Saharan Africa’. The search strings for each database including additional keywords searched are located in S1 Fig. A total of 3779 studies were obtained from the database searches.

After the removal of 998 duplicates in Covidence, two pairs of reviewers (RVB and PR, and RK and VS) each screened 1390 and 1391 titles and abstracts respectively [18]. When necessary, a consensus was reached through group discussion. If no consensus was reached, an independent reviewer was consulted to reach a resolution. References of included articles were searched to further verify the comprehensiveness of source retrieval; however, no new articles were found.

After 216 (9.9%) discrepancies were resolved in the title and abstract screening phase, the same two pairs of reviewers screened 153 full-text articles. An Excel sheet was formulated for full-text screening to confirm the inclusion criteria of primary articles, which consisted of: mention of COVID-19 impact on HIV healthcare delivery and service; and mention of female disaggregated data.

The Excel sheet aimed to extract pertinent details from the articles, encompassing key population demographics, intervention settings, demographic traits, and significant findings. Through meticulous examination, the reviewers looked at the study populations of each article. Through this review, various common key population groups were presented throughout the research data extraction: women living with HIV, female sex workers, pregnant women living with HIV, and facilitators. Additionally, reviewers extracted several key patterns within the healthcare services delivery sector that was encountered by one or more of the key populations, highlighting issues such as accessibility, affordability, and availability of HIV services.

**Fig 1:**
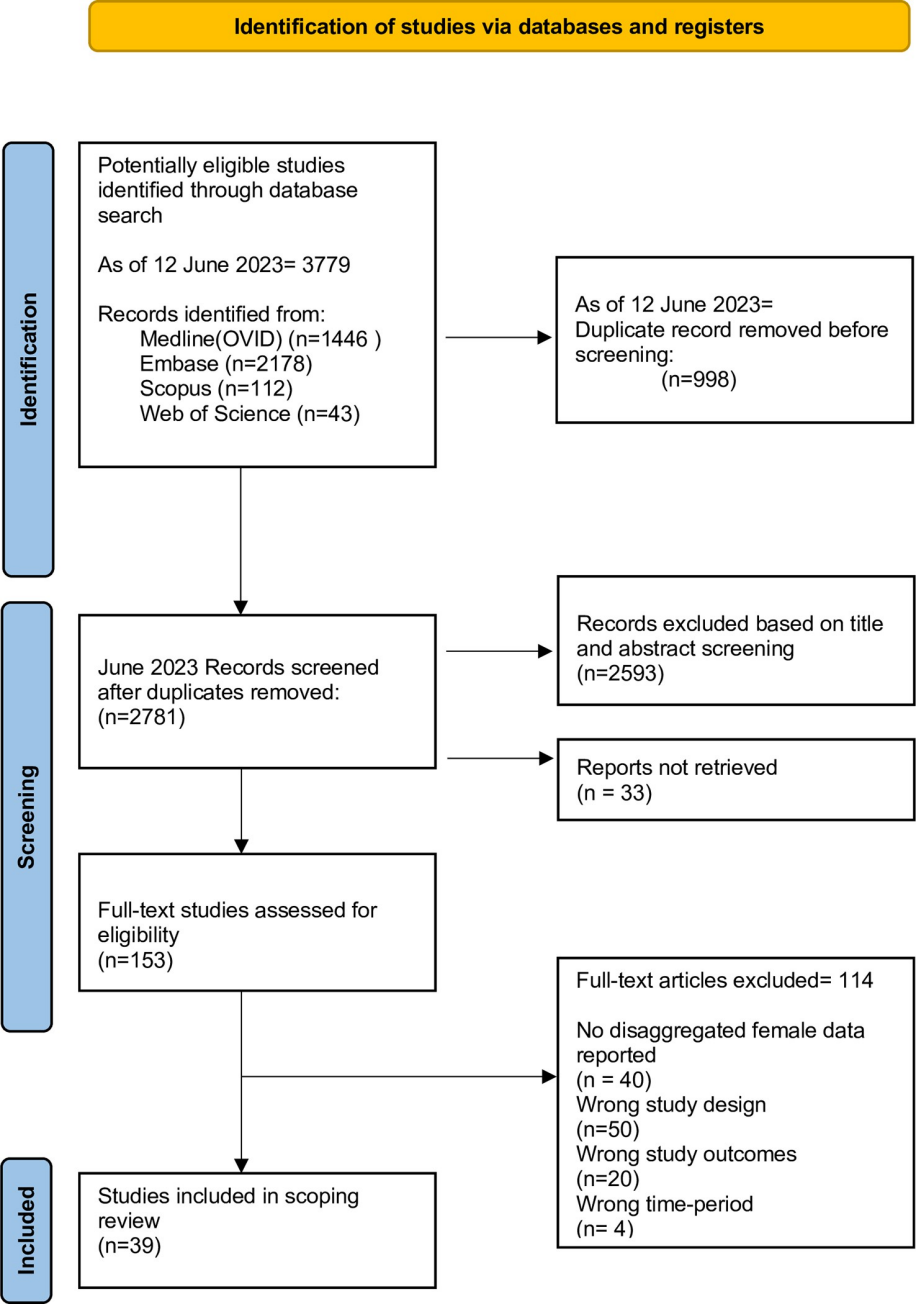
PRISMA flowchart. PRISMA flowchart of abstract and full-text screening results.

Articles were excluded at the full-text stage for the following reasons: no female-aggregated data was reported (34.5%), the study was not conducted during the COVID-19 pandemic (3.7%), the article did not include primary research (45.8%), and the study did not measure the outcomes of interest (15.9%) (Fig 1).

### Data extraction

Annotations of full-text articles were created to facilitate the data extraction phase and write-up process. All the lead authors extracted 10–11 articles each following data extraction criteria set for creating annotations. Included in the annotations were study design, data sources, population of the study, study time period, country of study origin, intervention, comparator, outcomes measured and results (S1 Table). Four study authors (RVB, RK, PR, VS) conducted a thematic analysis of the data extraction and annotations, which allowed for a flexible approach to interpreting the data. Each author independently identified topics with relevant supporting information from each included article. As a group, the authors then reviewed all of the identified topics and achieved a final set of relevant themes, sub-themes, and comprehensive definitions for each theme through analytical discussions.

The annotations helped to determine common themes among the included articles to help articulate the scoping review. Although a standard data extraction template was used, the summarization of the results found in the articles was subjective, which allowed researchers to compare views and extract shared meanings [19].

## Results

### Characteristics of included studies

**Fig 2:**
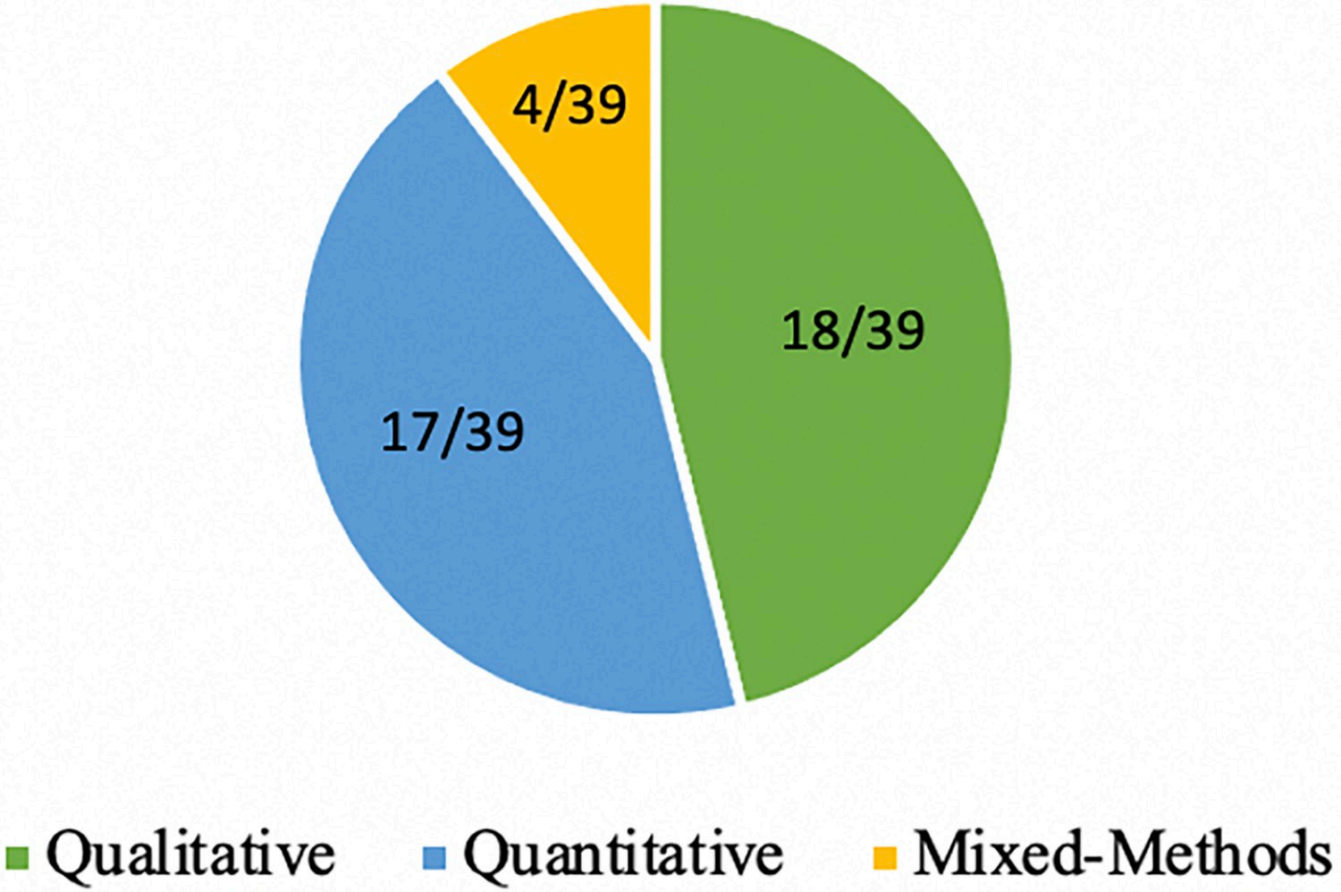
Study design of the included articles.

**Fig 3:**
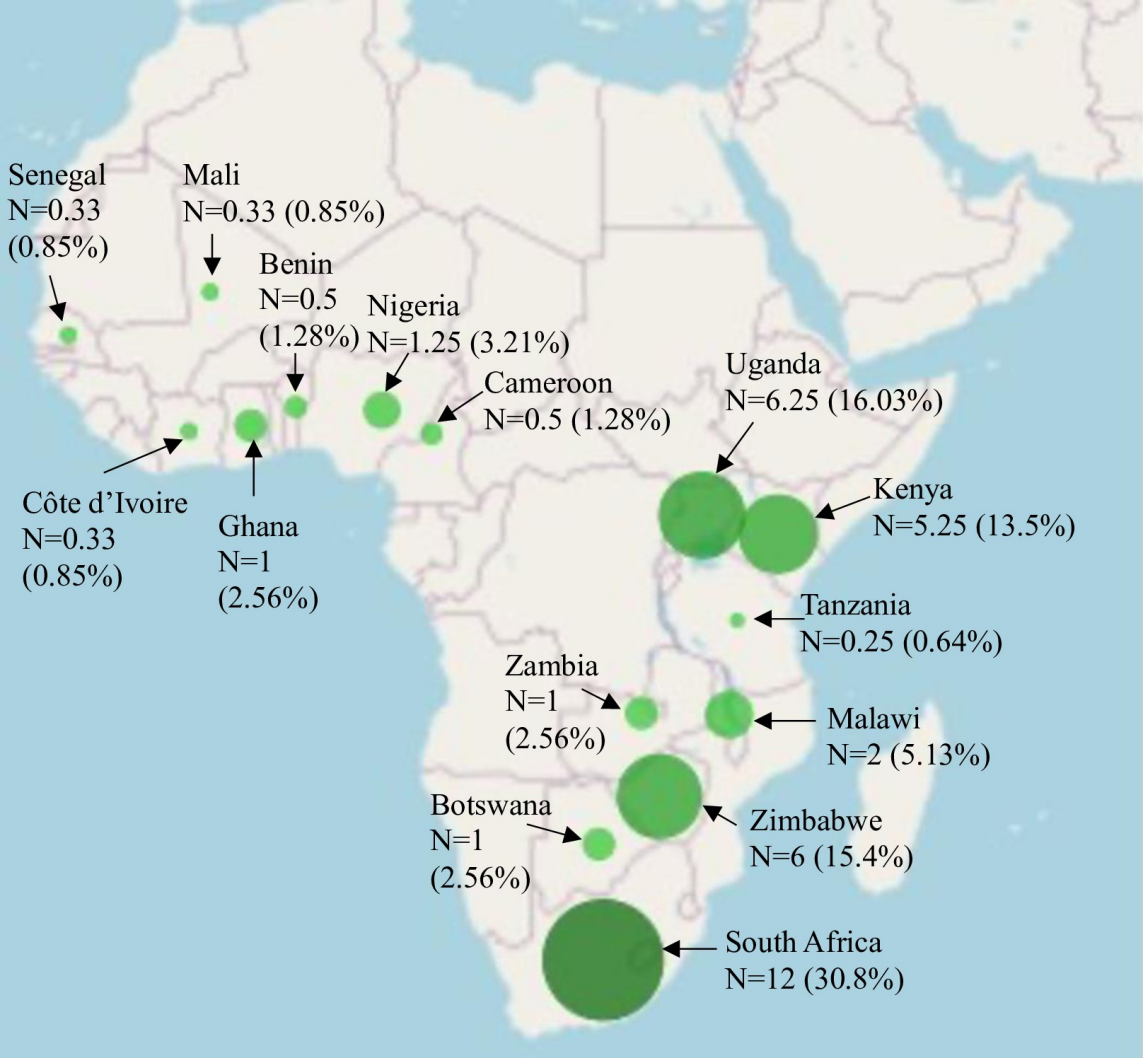
Study settings in sub-Saharan Africa (SSA) of the included articles.

**Fig 4:**
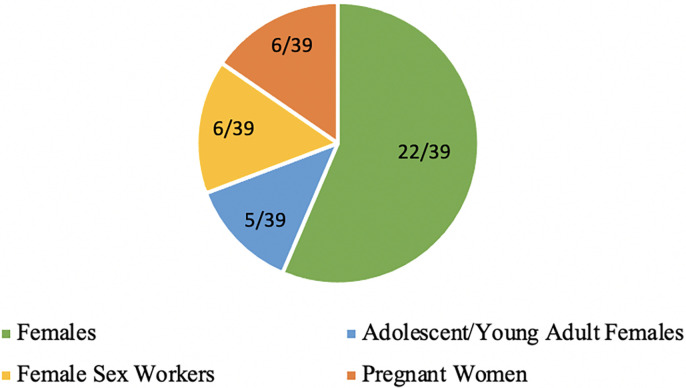
Population group studied in the included articles.

Of the 39 articles included in this review, 18 were qualitative, 17 were quantitative, and four used a mixed-methods study design (Fig 2). The included studies were from 16 different countries within SSA, with South Africa being the most frequent study setting (Fig 3) (S2 Table). Studies examined different population groups who were living with or at risk of HIV including adolescent/young adult females specifically (n = 5), female sex workers (n = 6), females who were pregnant or postpartum (n = 6), and females not otherwise a part of a sub-population (n = 22) (Fig 4). Several overarching themes were identified by the review authors from the included articles including: (1) accessibility, (2) availability, (3) affordability, (4) clinical barriers (5) psychosocial barriers, (6) special populations including female sex worker (FSW) and pregnant females, and (7) facilitators of HIV services and care (Table 1).

**Table 1:** Summary of themes and sub-themes.

Themes	Sub-themes	Main Lesson	Citations
Accessibility	PrEP, medication, general HIV services, and transportation	• Change in access to PrEP services during the COVID-19 pandemic significantly decreased PrEP uptake in female populations, resulting in a peer-led community-based program to increase PrEP uptake. • Lockdowns displaced females and decreased access to ARTs leading to more females participating in multi-month dispensing programs. • Access to HIV services experienced large disruptions for adult and youth female populations. • Decreased public transport routes, increased cost of transportation, and lack of understanding COVID-19 pandemic-related restrictions posed barriers to accessing ART clinics.	[21, 22, 24, 27, 29–34, 36, 38]
Availability	PrEP, medication, and HIV services,	• Shortages in PrEP and ART medications, and closure of school-based and NGO providers of HIV services further limited availability of HIV services for females.	[20, 29, 31, 38, 40]
Affordability	N/A	• Loss of income and additional clinic fees during the COVID-19 pandemic posed barriers for females.	[29–31, 35, 42, 43]
Clinical barriers	N/A	• Mistreatment by healthcare workers, long wait times at clinics, and reduced privacy at clinics posed barriers on females accessing HIV services.	[29, 31, 43, 44]
Psychosocial barriers	Stigma, and fear of COVID-19	• Fear of disclosing HIV status to a partner worsened during the COVID-19 pandemic and decreased female adherence to HIV medication. • Females feared COVID-19 and faced a dilemma of whether to place themselves at risk by going to HIV clinics, but ultimately COVID-19 did not drastically influence behaviour.	[29, 31, 35, 45]
Special populations	Female sex workers, pregnant females,	• Stigma and discrimination of female sex workers (FSW) carrying HIV was exacerbated during the COVID-19 pandemic when visiting HIV clinics, and dissuaded FSWs from visiting HIV clinics. • Female sex workers did not have access to support programs, resulting in decreased access to PrEP and ARV refills. • During lockdown, curfews were implemented, and HIV self-test kit distribution slowed from outreach groups to FSWs, decreasing testing until curfews were lifted. • Prevention to Mother to Child Transmission (PMTCT) program treatment re-location for migrant pregnant females living with HIV (FLH) was a main driver in the lack of adherence to HIV medication during the COVID-19 pandemic. • Border closures increased cost and complexity of accessing HIV treatment for pregnant FLH. • Lack of PMTCT education and counseling, and mistreatment by hospital personnel during the COVID-19 pandemic led to low treatment uptake by pregnant FLH.	[29, 42, 46–54, 56]
Facilitators	N/A	• Immunocompromised risk perception among FLH led to increased motivation to continue ART medication during the COVID-19 pandemic. • Delivery service of HIV medication, home-delivery, and multi-month prescription dispensing alleviated access burden of HIV medication for FLH during the COVID-19 pandemic. • Virtual care and community support increased PrEP uptake among FSWs during the COVID-19 pandemic.	[21, 33–36, 40, 42, 43, 57, 58]

A Python-generated map was created using Plotly, an open-source graphing library. The larger the circles represent a higher number of studies conducted. Studies settings include South Africa [18, 22, 25, 33, 37, 38, 40, 43, 48, 49, 52, 53], Uganda [19, 24, 28, 31, 36, 39, 50], Zimbabwe [17, 20, 30, 34, 42, 46], Kenya [23, 28, 29, 35, 41, 44], Malawi [8, 32], Nigeria [27, 28], Botswana [54], Ghana [26], Zambia [21], Benin [45], Cameroon [45], Cote D’Ivoire [47], Mali [47], Senegal [47], and Tanzania [28]. For studies that included multiple countries in SSA [28, 45, 47], the review authors counted each individual country as a fraction of the total number of countries investigated in the study. One study which looked at eight unspecified countries in SSA was not included in this figure.

### Accessibility

Among the articles reviewed, it was identified that females living with HIV (FLH) faced several additional barriers to accessing HIV services and medications due to the COVID-19 pandemic. For the purpose of this review, accessibility refers to females’ ability to use HIV care and services and the challenges faced while attempting to use them. Such challenges ranged from accessing PrEP and HIV medication, general HIV services, and transportation.

#### PrEP

Pre-exposure prophylaxis (PrEP) are effective preventative medications that reduce the risk of contracting HIV [20]. Prior to the COVID-19 pandemic, Zimbabwe reported up to 1600 PrEP initiations per month, but during the early stages of the COVID-19 pandemic, the number of initiations decreased to 408 per month [21]. It was suggested that this occurred because conventional methods of accessing PrEP were altered due to the COVID-19 lockdown [21]. Females in South Africa also reported confusion regarding changes to PrEP clinic hours because of the COVID-19 pandemic which decreased uptake [22]. By September 2020, with the roll out of successful peer-led and community-based programs in Zimbabwe, PrEP initiations spiked in the country to 1603 per month [21].

#### Medication

The COVID-19 pandemic resulted in the displacement of FLH, due to lockdowns and general pandemic restrictions [23, 24]. For example, a 49-year-old FLH using ART described the difficulty in acquiring medication as a result of the COVID-19 pandemic restrictions:

“I spent 2 weeks without taking medication as I was locked down in Bubi, where I had visited my sister. The local clinic told me that they had supplies for their registered patients only and advised me to go to my registered clinic which was impossible due to lockdown” [24].

Additionally, in South Africa and Kenya, changes in ART clinic operating hours due to the COVID-19 pandemic limited FLH ability to access ART, which decreased their adherence to life-extending medication [25, 26]. At the same time, participation in differentiated service delivery (DSD) of ART increased during the COVID-19 pandemic in areas where the service was not previously implemented [27]. Differentiated service delivery helps to improve access to HIV medication by extending dispensing intervals and/or increasing locations of service delivery [27].

#### General HIV services

Despite a general decrease in access to HIV medication during the COVID-19 pandemic, Palattiyil et al. (2022), in Uganda, found that community-based healthcare services were able to appropriately adapt to changing community regulations and improved access to HIV care for females [28]. Interestingly, in Nigeria, females with higher socio-economic status (SES) reported more limitations in access to HIV services than females classified in the middle SES within the context of the COVID-19 pandemic [29]. However, the reasons behind this finding were not explored. In contrast, Humphries et al. (2022), reported that in South Africa, a greater number of household members being able to contribute financially was protective against COVID-19 pandemic disruptions to HIV care [30]. Transgender females and FSWs in Nigeria and South Africa also experienced greater limitations to accessing HIV services than any other female population subgroup during the COVID-19 pandemic [29, 30]. Moreover, females of older age (>25 years) were reported to face fewer disruptions to HIV services compared to females between the age of 15 and 18 during the COVID-19 pandemic in South Africa [30]. The authors suggest the COVID-19-related disruption in school-based sexual health programs limited access to HIV services among younger females [30]. Abraham et al. (2022) reported that 50% of females in their sample population found difficulties in accessing HIV healthcare services during the COVID-19 pandemic [31]. Females were also more likely to miss HIV clinic appointments during the COVID-19 pandemic compared to pre-pandemic times [32]. All in all, it was clear that females across SSA experienced large disruptions to accessing general HIV services because of the COVID-19 pandemic.

#### Transportation

Transportation was repeatedly cited in the literature as a barrier to females across SSA accessing HIV services specifically during COVID-19 pandemic. This included a lack of public transit routes, the increased cost of transportation, and a lack of understanding of the COVID-19 pandemic-related restrictions imposed in some settings.

The COVID-19 pandemic resulted in some females relocating due to cross-border restrictions and for employment, which generated additional transportation challenges to FLH accessing HIV services [28, 33]. One study participant from Uganda reported that public transport was banned due to the COVID-19 pandemic, and when the ban was lifted, the costs became too high which made it difficult for them to access HIV treatment [28]. Some females in Zimbabwe were required to apply for travel permits to reach the hospitals at which they were registered for care [34]. The curfews and ban on public transport severely impacted the mobility of females requiring HIV services [35]. Moreover, some FLH reported abuse by police officers at COVID-19 restriction related roadblocks which limited their ability and willingness to travel to ART clinics [24]. Police and soldiers particularly lacked education on how to serve FLH during the COVID-19 pandemic, which negatively impacted their ability to guide FLH to alternate routes to their ART clinics [24].

Testing for HIV and other HIV-related services were also interrupted due to the COVID-19 pandemic restrictions because of the lack of transportation and closure of some community testing centres in Malawi and South Africa [36, 37]. Mobile HIV testing clinics became available, but fear of contracting COVID-19 also discouraged females from attending the mobile clinics and getting tested [36].

### Availability

This study defines availability as a measure of having HIV care, services or medication to meet the needs of the clients. During the COVID-19 pandemic, shortages in PrEP and ART medications emerged, which further exacerbated the disadvantages that females face to using HIV services [21, 29, 38].

#### PrEP

In Zimbabwe, females noted a decreased availability of PrEP medication specifically during the COVID-19 pandemic [20]. The reason for this is two-fold. First, there was a surge in the uptake of PrEP among the FSW population which resulted in an increased demand for the medication [20]. Second, there was a national shortage of PrEP medication when the COVID-19 lockdown restrictions were lifted [20].

#### Medication

During the COVID-19 lockdowns, ART clinics remained open and mobile message alerts were sent to clients to increase awareness of the services’ availability [31]. However, during the COVID-19 pandemic, medication supply chains were negatively impacted which limited the availability of ARVs for FLH [31]. Overtime, these barriers resulted in individuals and countries experiencing drug shortages. In Western Kenya, 52% of FLH reported concerns about running out of HIV medication [39].

However, females in Ghana continued to receive medication on time, just in lesser quantities or alternate medications were administered when necessary [31]. Some clinics adopted appointments for patients to obtain pre-packaged bundles of multiple medications to address co-morbidities, which decreased wait times for clients [40]. Moreover, some clinics improved availability by expanding their network to provide counselling at multiple sites [40].

#### HIV services

Females living with HIV experienced limited availability of HIV services specifically during the COVID-19 pandemic [29]. Prior to the COVID-19 pandemic, schools and local non-governmental organizations (NGO) would willingly provide HIV services; however, due to the COVID-19 pandemic, these services were closed [29]. In addition, with the sudden closures and lockdown, the availability of HIV social educational activities within health clinics were limited. Females living with HIV found it difficult to access services such as HIV testing and HIV self-screening kits during the COVID-19 pandemic [40]. Despite this, females continued to attend a community-based integrated HIV program for youth to access the HIV healthcare services they needed [38]. Door-to-door PrEP, HIV testing, and counselling were adopted in some communities in an attempt to address the barriers posed by the COVID-19 restrictions, and although this improved availability, many females did not use the services out of fear of contracting COVID-19 [37].

### Affordability

This study identified affordability of HIV services and medications as another theme within the literature. Affordability was defined as the financial aspects acting either as a barrier or facilitator of accessing HIV services or healthcare.

The COVID-19 pandemic worsened the economic barriers that females faced to accessing HIV services. For example, females over the age of 15 in Nigeria and Uganda, reported financial barriers which were exacerbated by the COVID-19 pandemic, including transportation costs, costs of medication or tests, fees at HIV clinics, additional unofficial clinic fees, and loss of income from the clinic visit [29, 40]. The largest financial barrier reported by the females in the study conducted in Nigeria was the additional cost of unofficial fees, followed by lost income from the hospital visit [29].

In Malawi and South Africa, females noticed that due to the COVID-19 pandemic, certain ART medications were unavailable at HIV clinics [41]. If the medication that had been prescribed was unavailable, some FLH encountered an additional cost to dispense a new medication [41]. This added financial strain impacted ART medication adherence [41]. Moreover, the limited ARV medications due to the COVID-19 pandemic resulted in some FLH having to travel to HIV clinics more frequently, which was an unexpected financial burden that limited the affordability of HIV medications [41].

Similarly, many ART clinics began to implement COVID-19 protocols such as mandatory masking [41]. The additional cost of this personal protective equipment (PPE) hindered females’ utilization of the HIV clinics, and some FLH were even turned away if they did not have the proper PPE [41]. Furthermore, females reported loss of employment because of the COVID-19 lockdown which decreased their ability to afford food [35, 42, 43]. The higher levels of food insecurity negatively impacted HIV medication adherence among FLH because of the extreme side effects when taking HIV medications on an empty stomach [28, 35, 41, 42].

### Clinical barriers

Clinical barriers refer to challenges experienced by FLH in healthcare settings (e.g., hospital, clinic, etc.). Several of the included studies identified barriers related to the healthcare environment that were unique or exacerbated due to the COVID-19 pandemic, such as mistreatment by healthcare workers (HCW), long wait times at clinics, and reduced privacy at clinics [29, 31, 43]. This mistreatment deterred females from seeking and receiving HIV care and services. Females living with HIV reported that they were treated poorly and humiliated by HCW at HIV clinics during the COVID-19 pandemic [29]. In the study by Kelly et al. (2022) one FLH reported being ignored and told by a HCW at the HIV clinic that because she did not have high priority health concerns, they could not provide her with the ARV medication she required [44]. The study by Abraham et al. (2022) similarly reported that FLH could not obtain refills of their HIV medications partially due to the higher patient volume at clinics during the COVID-19 pandemic [31].

The longer wait times at the HIV clinics and pharmacies resulted in FLH being required to wait outside, which made females less inclined to access HIV care [43, 44]. Additionally, the lack of adherence to COVID-19 protocols by healthcare staff, despite requiring patients to diligently follow them, created frustration among FLH and posed as a barrier to care [31].

Due to COVID-19 social distancing measures, FLH also reported reduced privacy at the HIV clinics, as their personal health information was spoken about in front of other individuals at the health center [43].

#### Psychosocial barriers

Psychosocial barriers refer to the combined challenges of psychological factors and the social environment that females at risk of or living with HIV experience and/or report. Several of the included studies reported on psychosocial factors specific to the context of the COVID-19 pandemic, which posed as barriers to females accessing and adhering to HIV services and treatment.

#### Stigma

One study reported that fear of HIV positivity status being revealed to their partner or other household members was worsened due to the COVID-19 pandemic lockdowns, which impacted the ability of FLH to adhere to their medication [35]. The COVID-19 lockdown procedures resulted in partners and household members often remaining in the same household together for extended periods of time. The fear that their partner and other household members would discover their positive status and the increased visibility among members of the same household during lockdowns prevented FLH from taking their HIV medication and resulted in missed doses.

#### Fear of COVID-19

Fear of being exposed to and infected with COVID-19 while traveling to or at HIV healthcare and testing centers was another frequently reported barrier to females to accessing HIV care and services [29, 31, 35, 45]. Females living with HIV understood that they were at a greater risk of experiencing worse health outcomes from COVID-19 infection because of their immunocompromised status compared to individuals not living with HIV and were fearful of contracting the virus [31]. Females living with HIV experienced the dilemma of potentially exposing themselves to COVID-19 by attending HIV clinics or not attending HIV clinics and placing themselves at greater risk of HIV-related health complications [34]. Despite this fear, Abraham et al. (2022) reported that only one in ten FLH from Ghana did not attend the HIV clinic for this reason [31]. Females living with HIV who had supportive family members reported that their family was able to attend the clinic on their behalf to obtain their HIV medication [31].

### Special populations

Several articles investigated specific female sub-populations who experienced unique barriers and facilitators to accessing HIV care and services as a result of the COVID-19 pandemic. As such, the authors recognized the need to highlight two groups, FSW and pregnant females living with HIV, as special populations.

#### Female sex workers

Female sex workers are defined as a ‘key population’ by the World Health Organization because they are more susceptible to being infected with HIV [46, 47]. Local laws, stigma, discrimination, and police harassment have contributed to FSWs vulnerability to HIV [46, 47]. Therefore, the study authors aimed to specifically explore FSWs access to HIV care and services. Female sex workers have experienced inequalities in accessing HIV services and care during the COVID-19 pandemic [29, 48, 49]. One study reported that FSW in Kenya were dissuaded from accessing the public ART clinics because of fear of contracting COVID-19, stigma that they carried the COVID-19 virus, aggression they experienced from police surrounding the clinic, and curfews imposed during the COVID-19 pandemic [48]. Furthermore, in Kenya during the COVID-19 pandemic, FSWs did not have access to the Bar Hostess Empowerment and Support Programme (BHESP) clinic, an organization for FSWs that includes health clinics and sex worker-friendly faculty [48]. The inaccessibility of this service resulted in FSWs not receiving the PrEP and ARV refilling they required [48]. Additionally, when FSWs were asked to wait outside health facilities for COVID-19 screening purposes, they reported fear of having their HIV positive status being revealed to clients [46, 47]. This could potentially jeopardize their work and ultimately, was a barrier for their care [46, 47]. Moreover, compromised healthcare services, discrimination, and lack of adequate HIV care resulted in FSWs expressing frustration with the HIV service delivery during the COVID-19 pandemic [46–48].

A community-based project in Malawi distributed self-test kits to FSWs during the COVID-19 pandemic [11, 50]. These outreach activities were based on small group interactive sessions within public spaces, involving home visits, and small group and large group discussions [48, 51]. However, curfews were implemented during the lockdown, in addition to closures of restaurants and bars, which decreased the number of test kits distributed per contact [48, 51]. Once the curfews were lifted, this resulted in a slow recovery to return to pre-pandemic levels of test kit delivery [48, 51].

#### Pregnant females

Pregnant FLH are commonly enrolled in the Prevention to Mother to Child Transmission (PMTCT) programs in SSA as part of their HIV support services. During the COVID-19 pandemic, many pregnant FLH faced additional barriers pertaining to transportation, medication, educational outreach, or quality of healthcare [50, 52–55]. Treatment re-location for cross-border migrants going into South Africa was a main driver in the lack of adherence to HIV medication by pregnant FLH [50, 52]. Because of COVID-19-related border closures, pregnant migrant FLH experienced difficulties navigating the healthcare system of an unfamiliar country [50, 52]. Border closures also limited the affordability of transport, resulting in pregnant FLH attending less clinic appointments [53]. Interprovincial and intraregional pregnant migrant FLH could not afford transportation costs to clinics because of employment termination during COVID-19 [50, 52]. The need for documentation at borders during the COVID-19 pandemic also prevented some pregnant FLH from accessing PMTCT services [50, 52]. One pregnant FLH from Malawi was arrested several times in a hospital because her passport expired while South Africa was under lockdown, and nurses did not want to provide her with PMTCT [50, 52].

In contrast, Mutyambizi et al. (2021) reported that PMTCT services in South Africa were not affected by the COVID-19 lockdown [56]. This may be, as the authors of this study propose, because patients tend to receive antenatal care and PMTCT while receiving other of healthcare services that were not impacted by the COVID-19 pandemic [54]. Furthermore, this study investigated the differences in antenatal care attendance between January to March, 2020 and April to June, 2020, as well as April to December, 2019 compared to April to December 2020, which may not wholly encompass the impact that pregnant FLH experienced during the entirety of the COVID-19 pandemic.

The COVID-19 pandemic increased HIV medication adherence among some pregnant FLH [50, 52]. Pregnant mobile FLH on ARVs were given multi-month dispenses during the COVID-19 pandemic. The pandemic increased the need to prescribe multi-month ARVs because of lockdowns and border controls [53]. In contrast, other studies conducted in different countries in SSA report how medication shortages resulted in HIV clinics providing medications to pregnant FLH for shorter durations, such as less than a month, to allow for widespread access [52].

There was a lack of PMTCT education and counseling during the COVID-19 pandemic, resulting in many patients not receiving the appropriate information about the service [50]. The lack of consultation rooms because of COVID-19 restrictions made the dissemination of information in confidential spaces more difficult [50]. Educational training of nurses on disseminating PMTCT information was disrupted due to the COVID-19 pandemic [50]. Pregnant FLH patients felt that HCW could have explained the process better, noting confusion surrounding the program [50]. Thus, the study recommended outreach to better educate interprovincial and intraregional migrants on the PMTCT services and increase their HIV adherence [50].

Negative experiences with the healthcare system during the COVID-19 pandemic also dissuaded pregnant migrant FLH from accessing PMTCT services. For example, pregnant migrant FLH reported mistreatment by nurses, including a lack of respect, verbal abuse, and sub-optimal care compared to local patients [51]. Pregnant migrant FLH reported negligence in labor wards, noting how one mother living with HIV gave birth to a child in a toilet, and how nurses would verbally abuse migrant females seeking PMTCT [50].

#### Facilitators

Although many of the included articles explored the barriers to HIV care and services, some articles also identified facilitators of HIV healthcare delivery during the COVID-19 pandemic and explored various ways to improve HIV healthcare delivery.

The COVID-19 pandemic increased the risk perception in FLH. A study by Linnemayr et al. (2021) in Uganda reported that FLH were aware that their HIV positive status may make them more susceptible to severe COVID-19 disease outcomes [35]. As such, many females in this study reported being motivated to continue their ART medication to minimize the potential health consequences of contracting COVID-19 [36]. Similarly, disclosing HIV status and the cessation of lockdown measures were also found to be facilitators of HIV testing and medication adherence [42, 57].

Furthermore, a method commonly used to improve access to HIV medications involved DSD and other similar community-based medication delivery programs. In Botswana, to decrease any additional burden on already strained healthcare facilities, a study looked at scaling up HIV medication pickup from private pharmacies and through home delivery specifically during the COVID-19 pandemic [58]. Within Botswana, acceptability of the home delivery method for HIV medication was high among females [58]. Similarly, the article by Nalubega et al. (2021) noted that in Uganda HIV treatment delivered to their home fostered continuity of care despite COVID-19 challenges [43]. In addition to home delivery, multi-month prescriptions of medications including PrEP also improved treatment adherence [21, 40].

Another common facilitator of HIV healthcare delivery during the COVID-19 pandemic was telemedicine. Two studies assessing the use of PrEP in FSWs adapted their study methods to include remote interviewing and physical distancing when picking up PrEP medication in Zimbabwe and Kenya respectively. Both studies noticed that these COVID-19-driven transitions to virtual and remote care improved female participant PrEP uptake; however, the reasons behind this were not explored [21, 33]. Virtual HCW and community support also improved overall adherence to PrEP for FSWs [21]. Similarly, other articles reported that supportive structures including peer educators on PrEP, peers that motivated females to overcome HIV stigma, and education on the health benefits of medication adherence perinatally, helped with HIV prevention and treatment adherence [21, 42].

## Discussion

This paper reviewed the literature on the impact of the COVID-19 pandemic on HIV healthcare delivery for females in SSA. This review identified several factors which were impacted and exacerbated specifically by the COVID-19 pandemic including accessibility, availability, and affordability of HIV testing, PrEP, medication, and transportation to HIV clinics among others. Additionally, this review revealed several clinical barriers, psychosocial barriers, and facilitators related to HIV care and services for females in SSA that emerged as a result of the COVID-19 pandemic. Female sex workers and pregnant FLH were identified as two special populations who experienced unique barriers to HIV care and service delivery during the COVID-19 pandemic. Each of the 39 studies included in this review reported challenges and disruptions experienced by females seeking HIV healthcare in SSA due to COVID-19.

### Implications

This study found that females in SSA experienced unique challenges in the availability, accessibility, and affordability of HIV care during the COVID-19 pandemic. Within the female population itself, FSW and pregnant females are key populations that require special considerations to tackle the unique barriers they face to HIV healthcare services, especially in the context of an unprecedented public health emergency such as the COVID-19 pandemic. As such, further research should focus on different female sub-populations living with HIV, which will allow for a more detailed exploration of the specific barriers faced by these groups during the COVID-19 pandemic and can inform tailored and more effective policies.

Based on the evidence synthesized in this review, the following recommendations were made to improve the provision of HIV healthcare to FLH for future emergencies and should be addressed by policymakers, healthcare administrators, and public health officials.

#### 1. Human immunodeficiency virus clinics should allow patients unregistered at their specific clinic to refill their HIV medication

Many FLH reported being unable to obtain HIV medication at the clinic they were registered at due to lockdowns, travel restrictions, and limited public transportation available [24, 28, 30, 33, 35]. Females living with HIV also reported being turned away from HIV clinics because they were not registered at that specific location. Therefore, HIV clinics should allow migrant FLH and other FLH who are unable to travel to the HIV clinic they are registered with to retrieve and refill their ART especially during public health emergencies and/or nation-wide lockdowns. Additionally, healthcare systems across SSA could strengthen existing health information communication systems to allow FLH to access their medications from any HIV clinic. This would not only improve the availability and accessibility of HIV medications but would also reduce cost barriers to obtaining HIV medications by reducing transportation costs and loss of income due to time taken for transportation to HIV clinics [29, 40, 50, 52].

#### 2. Differentiated service delivery of ART should be expanded

Differentiated service delivery participation increased during the COVID-19 pandemic and it is a useful HIV care model because it tailors the location of service delivery, frequency of clinic visits, and type of healthcare services received to the patient’s needs [27]. Therefore, policymakers should continue to develop and expand DSD in preparation for future public health emergencies. This can reduce the frequency by which FLH are required to travel to HIV clinics to refill their medication, which can reduce the financial barrier of transportation costs [31] and psychosocial barrier of exposure to potential pathogens such as COVID-19, which FLH reported being fearful of [32, 36, 37]. Home-delivery programs for HIV medication were also well-accepted during the COVID-19 pandemic in Botswana [58] and Uganda [43], and such programs are recommended to be expanded further during nation-wide emergencies in the future.

#### 3. Personal protective equipment should be provided to patients at HIV clinics

This review found that an important barrier to receiving HIV care was the cost of PPE and a lack of proper PPE available, which resulted in FLH being turned away from HIV clinics [31]. Ensuring that proper PPE is available for patients at HIV clinics can help protect both the patients and healthcare staff, and may help mitigate the commonly reported fear among FLH of contracting COVID-19 while at HIV clinics [29, 31, 35, 45].

Based on the evidence synthesized in this review, other recommendations to strengthen HIV healthcare delivery to FLH in SSA especially during public health emergencies include dispensing pre-packaged medication bundles at clinics to address patients’ co-morbidities [40]; expanding mobile HIV testing clinics [27, 36]; and developing and expanding community-based programs for vulnerable groups including youth, FSW, and pregnant FLH, which were proven to be feasible and well-accepted during the COVID-19 pandemic [11, 21, 38, 50].

### Future studies

Moreover, upon completing a review of the existing literature around the impact of COVID-19 on HIV healthcare delivery and use for females in SSA, several gaps were identified. To begin, one of the most common reasons for article exclusion during the full-text screening process was the lack of gender-disaggregated data. Overall, very few articles studied only females. Moreover, our review only identified five articles looking specifically at pregnant females. Pregnant females, as identified by this review, face unique barriers to accessing HIV care. Managing HIV in this population is of utmost importance because the virus can be transmitted from mother to child, leading to intergenerational HIV impacts [52]. Therefore, future studies on this topic should include gender disaggregated data or specifically focus on females and pregnant FLH, which are often under-researched population groups.

Similarly, another gap identified within the literature is the lack of representation of older aged females. Vertical transmission of HIV is only a concern for women of reproductive age, and as a result, older females living with HIV who are not of reproductive age are often an under-researched population. Older-aged females, due to their age-related immunosuppression, are disproportionately impacted by COVID-19 and may face unique barriers in accessing HIV care compared to younger females. Therefore, additional research should be conducted on the unique barriers that older females faced to accessing HIV care during the COVID-19 pandemic.

Future research should also explore evidence-based ways to ensure the continuation of HIV care amidst public health restrictions and lockdowns. The use of SMS-based interventions has been used to improve HIV care, but to our knowledge, this program’s applicability specifically during the COVID-19 pandemic has not been studied [59]. Furthermore, COVID-19 lockdowns resulted in an increase in gender-based violence and exposure to HIV stigma within households [29, 42]. Hence, future research needs to explore ways of protecting FLH currently and in preparation for future emergency lockdowns.

### Strengths and limitations

This scoping review provides insight into how the COVID-19 pandemic impacted the delivery of HIV healthcare and services for females in SSA. The methodological rigor enforced in the methods of this paper limited selection bias by using a double blind-system during the abstract and full-text screening stages. The inclusion of both qualitative and quantitative research strengthens the arguments made in this review by showcasing that the synthesized results can be encompassed by more than one study design. However, an overall lack of mixed-methods research or randomized control trial study designs was observed among studies, which may have limited the strength of the findings in this review. Moreover, many studies reported non-randomized sampling methods, which increase the bias in their findings and decrease the generalizability of the results. In addition, while many of the included quantitative studies provided evidence-based results, the rationales regarding changes to accessibility and availability of HIV services during the COVID-19 pandemic were not sufficiently explored, which limits the interpretation of their findings.

Many studies included in this review reported gender-disaggregated data, which typically do not focus on females specifically as much as articles which only include female participants. Many studies also did not distinguish whether they explored gender or sex, and reported small sample sizes; therefore, generalizability to females in SSA is limited. Lastly, there was a general poor representation of vulnerable and politically conflicted countries in SSA in the included studies, resulting in poor generalizability to all of SSA. Despite obtaining some abstracts of vulnerable and politically conflicted countries, the research did not develop into full-text published papers and were excluded from this review.

## Conclusion

This scoping review evaluated the impact of COVID-19 on HIV healthcare and service delivery for females in SSA. This review highlights the importance of considering and addressing the impact of public health emergencies and regulations on accessibility, availability, and affordability of HIV care for females. The results from our review suggest that there is an increased need for accessible, available, and affordable HIV healthcare and services for females in SSA, who are disproportionately impacted by HIV and COVID-19 healthcare disruptions. The research synthesized in this review should be used by HCWs at HIV clinics to gain a better understanding of the barriers experienced by females, which can improve the quality of care and services. Additionally, healthcare administrators, policymakers, public health officials, and the government should use this research to inform policies and guide decisions to improve equitable access of HIV services and care. Research on evidence-based strategies to mitigate this impact and maintain or improve HIV care for females in SSA during public health emergencies should be conducted to further guide these decisions.

## Supporting information

S1 Fig Search strategy strings. (TIFF)

S1 Table Data extraction sheet. (PDF)

S2 Table Distribution of study settings. Distribution of study setting by country in Sub-Saharan Africa for all included studies. Studies with multiple country settings received a fraction value based on the number of settings investigated in the study. One article, which studied eight non-specified Sub-Saharan African countries was not included in the data.
(TIFF)
