# Supplementary figures and images for: The impact of the COVID-19 pandemic on HIV healthcare delivery for females in sub-Saharan Africa: A scoping review
Source: PLOS Glob Public Health. 2024 Dec 5;4(12):e0002975. doi: 10.1371/journal.pgph.0002975 (PMC11620547; doi:10.1371/journal.pgph.0002975)

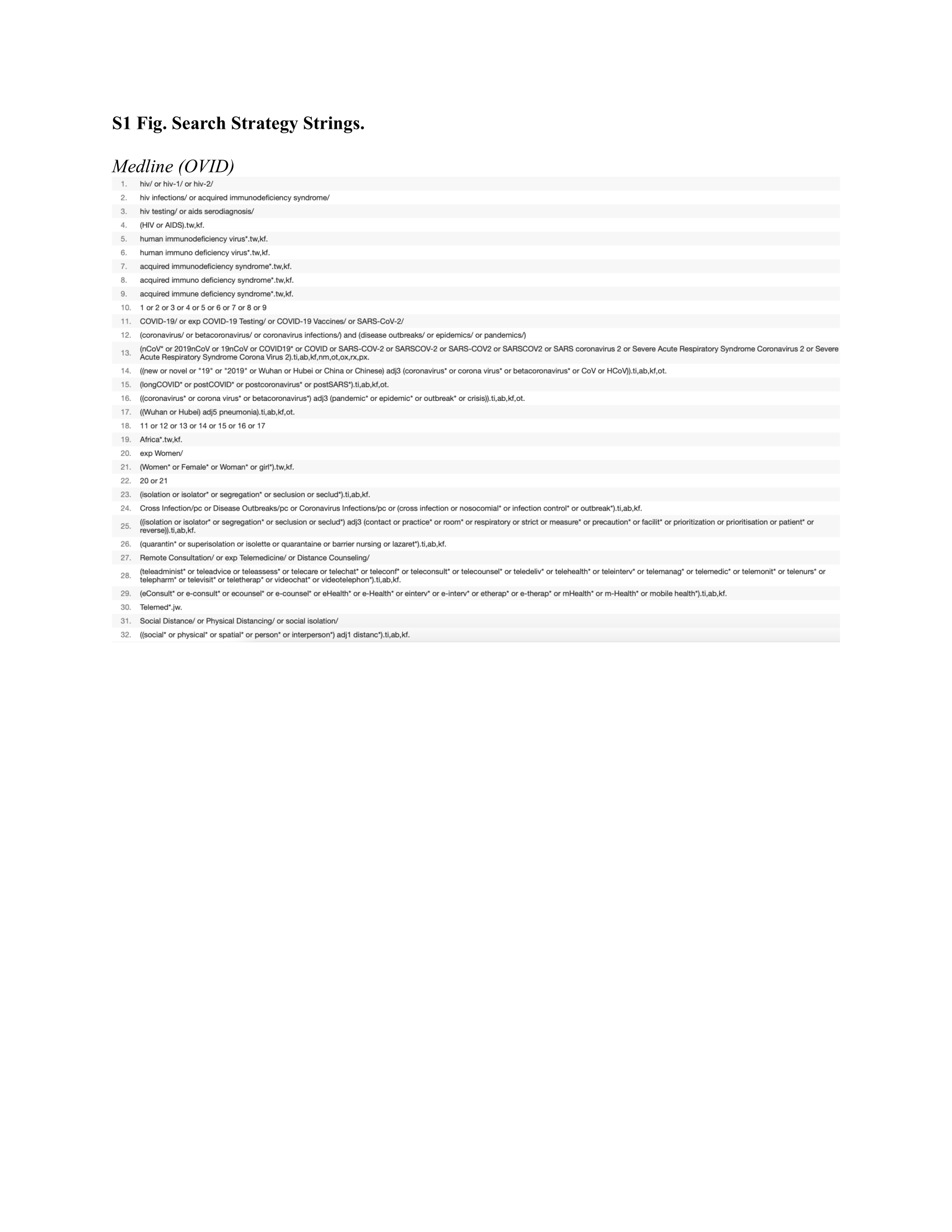

Supplement: S1 Fig — (TIFF) [file pgph.0002975.s001.tiff]

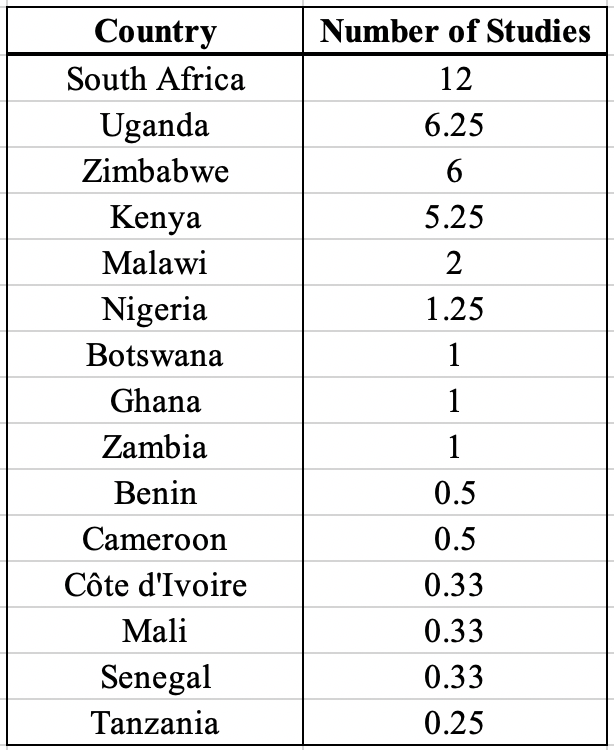

Supplement: S2 Table — Distribution of study setting by country in Sub-Saharan Africa for all included studies. Studies with multiple country settings received a fraction value based on the number of settings investigated in the study. One article, which studied eight non-specified Sub-Saharan African countries was not included in the data. (TIFF) [file pgph.0002975.s003.tiff]
