# Supplementary material for: The impact of the COVID-19 pandemic on HIV healthcare delivery for females in sub-Saharan Africa: A scoping review
Source: PLOS Glob Public Health. 2024 Dec 5;4(12):e0002975. doi: 10.1371/journal.pgph.0002975 (PMC11620547; doi:10.1371/journal.pgph.0002975)
Supplement: S1 Table — (PDF) [file pgph.0002975.s002.pdf]

S1 Table. Data Extraction Sheet

First Author	Year of Publication	Article Title	Study Design	Study Location	Year(s)	**Data Sources** (e.g., pt interviews, questionnaires)	Participants	**Intervention** (e.g., during COVID, post-COVID?)	**Comparator** (pre-COVID?)	**Outcome(s) Measured** (e.g., ART use, clinic visits, # tests)	Relevant Results
Abraham et al.	2022	Explorative-descriptive study on the effects of COVID-19 on access to antiretroviral therapy services: the case of a teaching hospital in Ghana	Qualitative Explorative study	Cape Coast, Ghana	January 2021- March 2021	Semi-strcutured patient interviews	18 years or older with at least 1-year hisotry of accessing care in the ART unit before COVID-19 and at least one clinic visit during the pandemic	During COVID-19 pandemic	Pre-COVID-19 Pandemic	ART clinic accessbility, ARV availabiltiy, afforability of treatment, accomodation	ART clinics did not close, but 1/10 participants did not use the clinics because fo fear of contracting COVID-19, 2/10 participants did not find ARV available during the pandemic, 1/10 participants found aquring PPE and medication challeging, 5/10 found access to healthcare professionals difficult, 3/10 participants noticed an increase in transportation costs, 2/10 participants defaulted with their refill medication. ART clinics remained open and sent messages to clients to make them aware of their constant availabiltiy. Narratives showed a general fear of contracting the virus because they are immunocomrpozied, yet only 1/10 did not go to the clinic because of this. People living with HIV that were afriad to go to the clinic had family suppor that picked up medication for them.There was a drug shortage, but people stil received medicaiton on time, just in lower quantities, and if some were unavailable alternative were arranged so no one would miss a dose. That made eople have to travel more to clinics, which increased transporttion costs. PPE was a cost that was required among people living with HIV to access ART clinics, which hindered some people's utilisation of the clincis. Additional dispending costs for medications not prescribed by the clinic were imposed on ART users, which affected their compliance. Patients were turned away if they did not wear proepr PPE. COVID-19 protocals needed to be stricly followed by participants, but caregivers were lenient themselves, which frustrated patients. Because of overcrowing at clincs, some people did not honour the refills. Longer waiting times created hesitation of whether or not to go to HIV clinics and ot pharmacies. Overall people were happy with teh healthcare delivery of HIV treatment during the pandemic.
Baker et al.	2023	Young people's access to sexual and reproductive health prevention services in South Africa during the COVID-19 pandemic: an online questionnaire	Qualitative study	Eastern Cape, South Africa	September - December 2020	Web-based questionnaire	Youth aged 15 to 24	During COVID-19 pandemic	Pre-COVID-19 pandemic	Acess to healthcare services	17.5% Females found it more difficult to access HIV testing services
Bernard et al.	2022	Impacts of the COVID-19 pandemic on access to HIV and reproductive health care among women living with HIV (WLHIV) in Western Kenya: A mixed methods analysis	Mixed methods	Kisumu County, Western Kenya	March to November 2019	Telephone survey	WLHIV (Chaguo Langu) 15 to 49 years of age	Post-COVID-19 pandemic	Pre-COVID-19 pandemic	Acess to HIV service and treatment	Access to HIV care and ART was interrupted in terms of retrieving medication refills (32%) and HIV care and services (14%), Younger people and female faced several fears and difficulties in accessing clinics during COVID-19. The feeling of overwhelm limited young peoples access to sexual and reproductive health services. resulting in high risks for HIV contexts and fear of expenses in which young people were unable to afford for these services.
Bisnauth et al.	2022	Addressing the migrant gap: maternal halthcare perspectives on utilising prevention of mother to child transmisson (PMTCT) services during the COVID-19 pandemic, South Africa	Qualitative study	South Africa	June 2020-June 2021	Semi-strcutured patient interviews	Migrant women of reproductive age of 18 years or older, HIV-positive, pregnant, newly delivered, or in post-natal follow-up	During COVID-19 pandemic	Comparison made among participants during the COVID-19 pandemic pandemic	Adherence and barriers to PMTCT services; treatment interruption, fear. Availability: space, PMTCT education and counselling. Affordability: job loss, documentation. Acceptability: mistreatment. Adaptability; same day initiation, individualised apporach.	Patients felt that healthcare workers could have explained the process better, a lot of unknowns. COVID-19 made pregnant women adhere to their HIV medications more. Treatment re-loation for cross-border was a main dirver in lack of adherence to HIV medication. Many interprovincial migrants interrupted their adherence to ARVs because of fear fo COVID-19 aroudn healthcare settings. Lack of consultation rooms because of social distancing protocols. Clinics are small, sanitary space was of concern for corss-border migrants whose home was far away. There was a large lack of PMTCCT education and counselling, so most patients were not well-informed, somefound educaiton outside of capitals better. Because of border closures, cross-border migrants has a harder time navigating the healthcare system of an unfamialr place. Interprovincial an dintraregiosnla migrants could not afford transportation cotss to clinics because o losing their job during COVID-19. The need of border documention during COVID-19 prevented some people from accessing PMTCT srvices. A pregnant women from Malawi was arrested serveral times during the pandemic because her passport expired while South Africa was under lockdown, and nurses did not want to treat her with PMTCT. There was a lot of negligence in the labour ward, one moter gave birth to a child in a toilet. Verbal abuse was endured my migrant women seeing PMTCT. Outreach is recommended for interprovincial and intraregional migrants to better educate them on the PMTCT services.
Bisnauth et al.	2022	Providing HIV Prevention of Mother to Child Transmission (PMTCT) Services to migrants during the COVID-19 pandemic in Aouth Africa: Insights of Healthcare providers	Qualitative study	South Africa	December 2019-undefined 2020	In-depth interviews	Healthcare providers reporting on females	During COVID-19 pandemic	Comparison made among participants during the COVID-19 pandemic	Adherence, availability, affordability, acceptability, adaptability	Mobile patients on ARVs were given multi-month dispenses during COVID-19, and the mothers were no longer going to clincis for th child immunizations, just to get medication each month. The pandemic increased the need to prescribe multi-month ARVS because of lockdowns and broder controls. Patients were scared to go to the hospitals ebcause they were afraid of getting COVID-19. Clinics that deliver babies ere closing, causing pressure on ones that were open. A booing system was put in place during COVID-19. Risk falling ill from brastfeedign during OVID-19 was high because of overcrowding. Educational training of nurses on disseminating infromaiton on PMTCT was stopped in the pandemic. Border closures created limited affordability of transport, so less appointments with the clinic. Nurses mistreated migrant patients with lack of respect and verbal abuse. Differences in care among cross-border migrants and locals. Same day initiation: patients received HIV medication the same day they were notified of having it. Referral systems became online platforms. Long queues and waiting lines decreased ART adherence among patients.
Burt et al.	2021	Indirect effects of COVID-19 on maternal, neonatal, child, sexual and reproductive health services in Kampala, Uganda	Quantitative Observational study	Kampala, Uganda	July 2019 to December 2020	Retrospective electronc medical rescord data	Patient reords of females	During COVID-19 pandemic	Pre-COVID-19 Pandemic	Pregnancy services, labour and delivery, availabiltiy of medicines	During lockdown, the proportion of women receiving HIV testing in antenatalcare declind by 4%. Patients receiving PMTCT decreased during COVID-19. Medication shortages affected the ability to give out ARTs during COVID-19.
Dear et al.	2021	Transient redutions in human immunodeficiency virus (HIV) clinic attendance and food security during the coronavirus disease 2019 (COVID-19) pandemic for people living with HIV in 4 African countries	Quantitative Prospective cohort	Tanzania, Uganda, Kenya, Nigeria	Januray 1, 2019 to March 2020 and May 7, 2020 to Februry 28, 2021	Biannual follow-ups and questionnaires	Disaggregated PLWH	During COVID-19 pandemic	Pre-COVID-19 Pandemic and people living without HIV	Missed HIV clinic visits	PLWH who missed clinic visits were more likely to be female (61.4% missed). Adherence was simialr to controls (people living without HIV)
Dorward et al.	2021	The impact of the COVID-19 lockdown on HIV care in 65 South African primary care clinics: an interrupted time series analysis	Quantitative Interrupted time seiries	South Africa	January 1 2018 to July 31, 2020	Prospective data set from 65 primary clinics	Disaggregated people living with HIV	During COVID-19 pandemic	Pre-COVID-19 Pandemic	People testing for HIV, initiating antiretroviral therapy (ART), and collecting ART at participating clinics during the study period	More females were testing for HIV post-lockdown, more femaless were starting ARt treatmetn post-lockdown, decreased risk for women adhering to ART collection visits post-lockdown, and decreased risk for missed ART collection visits.
Duby et al.	2022	Adaptation and Resilience: Lessons Learned from Implementing a Combination Health and Education Intervention for Adolescent Girls and Young Women in South Africa During the COVID-19 Pandemic	Qualitative study	South Africa	November 2020 to March 2021	Semi-strcutured individual interviews	Adolescent girls and young women	During COVID-19 pandemic	Pre-COVID-19 Pandemic	HIV testing and PrEP intake/ adherence	Enrollment into HIV educational programming for youth girls andwomen was limited in recruitment because of COVID-19 restructions, schools closed to visiors, children no longer on the streets, parents refused to give their child autonomy, HIV testing and PrEP dissemination were disrupted during the pandemic, no community testing allowed, only could have people go to clinics, postponed services. Mobile clinics opened up later on, but people mistook it for a covid-19 testing centre and were afriad to approach it due to fear of infection and wait-times. Quarantine disrupted uptake. Interviews had to be telephone interviews, but many did not have access to a phone or data, or were not comfortable talking abuout sensitive information ovr the phone. Door-to-door PrEP, testing, and counselling was adopted, many people rejected door-to-door service out of fear of COVID-19.
El-Krab et al.	2022	Subjective well-being and COVID-19 prevnetion practices among people living with HIV in Cape Town, South Africa	Qualitative study	South Africa	August to November 2020, no lockdown	Interview and survey	Disaggregated PLWH using a capetown clinic services	During COVID-19 pandemic	Pre-COVID-19 Pandemic	COVID-19 disruption on HIV care, and ART aherence	Women experienced disruptions in ART adherence during COVID-19, a lot of clnics were closed during the pandemic, a high number of women were unable to get to a pharmacy becase of COVID-19 ** don't show pre-covid numbers
Emmanuel et al.	2022	Utilization of ART services among people living with HIV during the COVID-19 pandemic: a case of Kampala district	Quantitative Cross-sectional study	Kampala, Uganda	March 24 to June 1, 2020	Electronic medical files and 647 patient's records followed prospectively from the clinic	Disaggregated PLHIV	During COVID-19 pandemic	Pre-COVID-19 Pandemic	ART utilization	ART utilization was inadequate for 14.71% of the female patients followed at the clinic, and 85.26% was good. The decrease is likely due to the COVID-19 restrictions.
Flanagan et al.	2022	Do not forget the children: a model-based analysis on the potential impact of COVID-19 associated interruptions in paediatric HIV prevention and care.	Quantitative Projection using Spectrum modelling package and the CEPAC-Pediatric model	Eight sub-Saharan African countries	September 2020 to March 2021	CEPAC-P, which was populated using cohort data from the International Epidemiology Databases to Evaluate AIDS East African cohort.	Pregnant women living with HIV	During COVID-19 pandemic	Pre-COVID-19 Pandemic	Number of women engaged in PMTCT care, new paediatric HIV infections, Children living with HIV (CLWH) receiving ART and death among all CLWH.	After 3 months of COVID-19 disruptions, mothers receving PMTCT had service disruptions.
Folayan et al.	2022	Factors associated with poor access to HIV and sexual and reproductive health services in Nigeria for women and girls living with HIV during the COVID-19 pandemic.	Quantitative - Cross-sectional	Nigeria	June, 2021 - October, 2021	Online questionnaire	Women and girls (age 15+) living with or at risk of HIV	1st year of the COVID-19 pandemic	N/A	- Access to HIV services: did the COVID-19 pandemic impact their attendance at health facilities for HIV prevention, treatment & care-related services (yes, no, not needed)<br>- Whether non-financial reasons prevented them from accessing HIV services	- 64.5% reported poor access to HIV services<br>- 15-24 year old women/girls living with HIV had significantly higher odds of reporting limited access to HIV services during the pandemic compared to those aged 25-44<br>- Participants with higher socioeconomic status had signficantly higher odds of reporting limited access to HIV services compared to middle SES<br>- Transgender women & women who sell sex had higher odds of reporting limited access to HIV services<br>- Women/girls with HIV with limited access to HIV services identified payment of additional unofficial fees, loss of income from hospital visits, having no money, fees at clinic/hospital, transportation costs & costs of medicines/tests were financial factors associated with poor access to HIV services during COVID<br>- Women/girls with HIV who had limited access to HIV services had significantly higher odds of reporting being humiliated at the last visit for HIV services, facing improper treatment at last visit, having HIV services usually offered at school/local NGO closed due to COVID, concern with being infected with COVID at health facility & risky road to health facility
Gichuna et al.	2020	Access to Healthcare in a time of COVID-19: Sex Workers in Crisis in Nairobi, Kenya.	Qualitative	Kenya	April, 2020 - May 2020	Interviews via mobile phone	Female sex workers living in informal settlements	During the COVID-19 pandemic	N/A	- Access to healthcare for sex workers<br>- Movement restrictions<br>- Social distancing measures<br>- Sexual & reproductive health access	- 1 sex worker was locked out of Nairobi & unable to access health services<br>- Cessation of movement in & out of areas in Nairobi (partial lockdown) --> no access for sex workers to BHESP clinic or sex worker-friendly facility<br>- 1 sex worker forced to go without PrEP, another couldn't get ARVs refilled<br>- Sex workers neglected HIV prevention & treatment services out of fear of catching COVID<br>- Dusk to dawn curfew was a barrier to healthcare access for sex workers<br>- Sex workers reported increased waiting time at healthcare facilities --> deterred access (some pts would be impatient & leave, some felt avoided/rejected)
Humphrey et al.	2023	Effects of the COVID-19 pandemic on late postpartum women living with HIV in Kenya	Quantative Study	Kenya	March 2018 to Feb 2019	Phone surveys	WHIV 18 to 24 months postpartum enrolled in HIV care	Post-COVID	Pre-COVID-19 pandemic	HIV services/medications	96% had adequate access to ART treatement, 6.5% concerned about running out of ART and 7.1% skipped ART dosage due to running out of treatment, 47% WHIV experienced income loss and 71% experienced food insecutiry
Humphries, H	2022	Impact of COVID-19 public health responses on income, food security and health services among key and vulnerable women in South Africa.	quantitative study	South Africa	Sept to Nov 2021	Surveys	Women over the age of 15 who self-reported living with HIV or at high risk of HIV (sex workers, adolesent girls and young women, LGBTQ+ women, migrant/refugees, women living with disability)	During COVID	Pre-COVID-19 pandemic	Access to HIV services, HIV family planning services	1167 women reported to be HIV+ (37% reported pandemic related disruptions to HIV services)<br>- sex wokers at higher risk of disruptions compared to non-sex workers<br>- Older age was protective with 25 years old being more protective compared to 15-18 years old<br>- Living in an informal housing, other forms of housing and traditional housing was more related to disruptions compared to house/flat<br>- Having more people in household contribute financially is protective, while alcohol abuse was disruptive<br>- Living in Western Cape compared to KZN and living in urban setting assocaited with more disruptions<br>- Highest disruptions reported among HIV positive sex workers, drug users, LGBTQ+ women, and adolesent girls and young women (belonging to more than 1 key vulnerable population = greater disruptions)<br>- adolesent girls and young women and sex workers reported highest disruptions in access to family planning services<br>- migrants (self-identified) and HIV positive sex workers at highest risk for food insecurity

First Author	Year of Publication	Article Title	Study Design	Study Location	Year(s)	**Data Sources** (e.g., pt interviews, questionnaires)	Participants	**Intervention** (e.g., during COVID, post-COVID?)	**Comparator** (pre-COVID?)	**Outcome(s) Measured** (e.g., ART use, clinic visits, # tests)	Relevant Results
Jo et al.	2021	Changes in HIV treatment differentiated care uptake during the COVID-19 pandemic in Zambia: interrupted time series analysis	Quantitative - Interrupted time series analysis, restrospective review	Zambia	September 2019 - August 2020<br>**Before**: September 2019 - February 2020<br>**After**: March - August 2020	Enrolment in differentiated service delivery models via Zambia's national electronic medical record system	Health facilities	During the COVID-19 pandemic (March - August 2020)	Pre-COVID-19 pandemic (September 2019 - February 2020)	- How differentiated service delivery model enrolement changed before & after start of COVID pandemic<br>- Uptake of models: fast track (<2 months, 3 months, 4-6 months), multi-month dispensing (3 months, 4-6 months), community adherence group, home ART delivery, others	- All models (proportion change % between before & after periods): 27% +<br>- <2-month fast-track dispensing: 76% +<br>- 3-month fast-track dispensing: 17%<br>- 4- to 6-month fast-track dispensing: -20%<br>- 3-month MMD (multi-month dispensing): 102%<br>- 4- to 6-month MMD: 26%<br>- Community adherence groups: 34%<br>- Home ART delivery: 142%<br>- Others: -15%<br><br>- Found overall increase in DSD (differentiated service delivery), but uptake varies widely by model<br>- While 4-6 month fast-track declined, 4-6 month MMD (multi-month dispensing) increased because of greater DSD enrolment in rural areas where fast-track is rarely implemented<br>- Participation in DSD models accelerated over study period (COVID pandemic associated with accelerated participation in DSD models in Zambia)<br>- NOTE: findings are based on patients NEWLY enrolling into a differentiated service delivery model (their 1st interaction with a DSD model only)<br>- Introduction of COVID-19 pandemic was associated with an acceleration in scale-up of differentiated service delivery models for clients on ART in Zambia
Kalua et al.	2022	HIV suppression was maintained during the COVID-19 pandemic in Malawi: a program-level cohort study	Quantitative - Population-based cohort study	Malawi	July 2019 - December 2020	Viral load measurement data from Malawi Laboratory Management Information System	Viral load samples from 556,281 patients at ART clinics	During the COVID-19 pandemic	Pre-COVID-19 pandemic	- Virologic suppression (viral load)	- Adjusted odds ratio for missed viral load among females was greater during COVID-19 pandemic than before COVID-19 pandemic
Kelly et al.	2022	HIV and SRH healthcare delivery experiences of South African healthcare workers and adolescents and young people during COVID-19	Qualitative	South Africa	Interviews with Healthcare Workers: August, 2020 - November, 2020<br>Interviews with Adolescents and Young People: April, 2020 - November, 2020	Semi-structured interviews	Registered professional nurses and adolescents & young people age 17-29	During the COVID-19 pandemic	N/A	- Barriers to accessing sexual and reproductive health & HIV services<br>- Addressing adolescents' healthcare needs	-Young people noted longer health facility waiting times as a barrier to service access - reported by female living with HIV<br>- Discomfort having to queue outside health facilities as a barrier, having to wait outside clinic<br>- Female reported poor treatment by healthcare workers<br>- 1 female reported the time & financial implications of being ignored at a busy clinic during COVID & was told that other patients were priorities --> couldn't get ARVs & was forced to skip them (they would tell her to come back another time, but treatment is finished, they need to help emergency people)<br>- At health care facilities, healthcare workers reported that they continued to offer regular adolescent services that were available before the pandemic (e.g., HIV monitoring, medicines pick-up, contraceptive services)
Kra et al.	2021	Introducing and Implementing HIV Self-Testing in Cote d'Ivoire, Mali, and Senegal: What Can We Learn From ATLAS Project Activity Reports in the Context of the COVID-19 Crisis?	Mixed methods	Cote d'Ivoire, Mali, Senegal	August, 2019 - December, 2020	Quantitiative & qualitative program data from HIV self-testing distribution. Focus groups and individual interviews.	Female Sex Workers	Initial emergency response (March - May 2020) to COVID-19 pandemic and the epidemic management stage (since June 2020)	Pre-COVID-19 pandemic (August 2019 - February 2020)	- Distribution of HIV self-testing kits<br>- Assessment of HIV outreach activities	- August 2019 - December 2020: 105,788 HIVST kits were distributed by the ATLAS project to FSWs<br>- Cote d'Ivoire: outreach activities targeting FSW were based on small group talks in public spaces<br>- Mali: several activities conducted to reach FSW (home visits, small group & large group activities)<br>- Cote d'Ivoire: closure of bars/restaurants & curfew --> decreased # of clients for FSW --> decline in avg # of HISVT kits distributed per contact<br>- Curfew lifted --> slow recovery & return of FSW pre-covid levels of test kit delivery<br>**Outreach activities:**<br>- **Cote d'Ivoire:** Adaptation of activities (March - May 2020)<br>- From public to private spaces, group size reduction, night activities moved to daytime, rapid tests converted into assisted HIVST, appt by phone/whatsapp, hygiene measures (COVID awareness, PPE, physical distancing)<br>- **Mali:** Adaptation of some activities (March - May 2020, region dependent)<br>- From public to private spaces, group size reduction, night activities moved to daytime, rapid tests converted into assisted HIVST, appt by phone/whatsapp, hygiene measures<br>- **Senegal:** suspension of outreach activities (March - May 2020) & resumption of activities (June 2020)<br>- Prioritization of face-to-face activities, less HIVST distributed per contact, appointment by phone/whatsapp, hygiene measures<br>- Gives quotes from female sex worker peer educators: "Before COVID, we used to go out at night to distribute to bars and restaurants. But with the pandemic and the restrictive measures taken on that occasion, we were obliged to change our strategy and give priority to home visits."
Linnemayr et al.	2021	HIV Care Experiences During the COVID-19 Pandemic: Mixed-Methods Telephone Interviews with Clinic-Enrolled HIV-Infected Adults in Uganda	Mixed-methods	Uganda	No information.	Telephone interviews	Ugandan adults on ART	During the COVID-19 pandemic.	N/A	- How the COVID-19 pandemic & lockdown affects access to HIV services & ART adherence<br>- Perceived effects of lockdown on HIV clinic attendance<br>- Perceived COVID lockdown effects on ART adherence	**Inability to continue ART visits due to transport, violence & money:**<br>- 1 female reported that they banned public transport, she could walk to clinic but there's curfew, once you get walking they beat you up<br>- Another female reports lack of transportation, can't get medication (last clinic visit 2-3 months ago)<br>**High Risk Travelling to Clinic:**<br>- Women concerned that other ART clients could be exposed to COVID during travel to HIV clinic --> greater risk to all clients on arrival<br>- Using public transport exposes to COVID<br>**HIV vs COVID Risk Dilemma:**<br>- Skipping HIV clinic visits (increase susceptibility of AIDS complications) vs attending the clinic (potential infection risk)<br>**COVID-19 effects on ART adherence:**<br>- Some female patients (2) reported no change in ART adherence even if the COVID situation is bad<br>- BUT, one women stated missed doses due to side effects of having to take ART without food (given lost employment & delayed government rations), another stated missed doses due to greater visibility among other locked down household members (missed doses due to potential unintended HIV disclosure to other household members)<br>- 1 female reported that ART adherence was motivated by its perceived potential to minimize infection/harm from COVID (if she takes ART medication well, body will be strong enough so if she gets COVID, she won't be as affected compared to if she didn't take ART)<br>- Several females aware that becuase they have HIV, COVID could affect them more than HIV-negative & should take greater care to protect themselves
Mackworth-Young et al.	2022	"Other risks don't stop": adapting a youth sexual and reproductive health intervention in Zimbabwe during COVID-19	Qualitative - Process evaluation	Zimbabwe	March, 2020 - September 2020	Interviews with providers, interviews with clients, non-participant observation of CHIEDZA sites, participant observation of regular CHIEDZA study team meetings	Healthcare providers, and CHIEDZA clients and healthcare providers	**Chiedza intervention** (community-based integrated SRH & HIV intervention for youth aged 16-24 aiming to improve population-level HIV viral load suppression)	N/A	- Participation in Chiedza sexual and reproductive health and HIV intervention	**- Sudden closure - disrupted access to HIV services** (due to government-mandated lockdown)<br>- Several females reported being able to ask questions, seeing people her own age, & doing other social activities, which were important to have so people attend and don't think it's just all about HIV testing<br>- Women continued to attend CHIEDZA to access much needed services (but this wasn't the same for men who didn't come just to get condoms) **NOTE services here could be referring to SRH or HIV
Mantell et al.	2021	Life in the Balance: Young Female Sex Workers in Kenya Weigh the Risks of COVID-19 and HIV	Qualitative Study	Kenya- Kisumu	Enrolled participants starting Oct 2019 to Feb 2020, 3 and 6 month follow up April and July 2020	Interviews	18-24 year old female sex workers and enrolled in the IPrEP study	During COVID-19 pandemic, participants were asked COVID-19 awareness and precautions questions, access to health services and their sex work experience during COVID-19. Baseline, 3 and 6 month follow up visit	N/A	Ability to obtain health care services, PrEP medication from the study, violence from a client or regular/main sexual partner, COVID-19 awareness/precautions	- 82.9% of participants indicated that COVID-19 did not impact their ability to obtain health services.<br>- 91.2% had no problem getting their PrEP medication<br>- Most common reasons for difficulty obtaining prep: moving out of study area, travelling to pick up meds<br>- HIV self-test kit results were reported to staf through SMS, in-person, photo through phone<br>- 44% of participants do not have enough money for essentials<br>- 45.1% report enough money for food but not much else<br>- 78.2% used a condom with last paying partner<br>- The study team modified their procedures in response to the pandemic to allow physical distancing and continued access to PrEP. Modifications including remote interviewing)<br>-The study reports that findings may not be generalizable to sex-workers that are not study-enrolled
Matambanadzo et al.	2021	"It went through the roof": an observation study exploring the rise in PrEP uptake among Zimbabwean female sex workers in response to adaptations during Covid-19	Quantitative study	Zimbabwe	Jan to Dec 2020	Clinical data from the *Sisters* clinic	Female sex workers	Changes to the Sisters with a voice PrEP framework- 1) trained to become advocates for PrEP within Sex work communities/ community-based PrEP services, 2) use of telehealth scaled up (WhatsApp adverse event reporting), 3)on-going virtial support - talk time and WhatsApp broadcast lists 4) supply 3 months PrEP at a time	These changes were made to adjust for COVID-19 lockdown and compare to Pre-COVID	HIV testing, PrEP screening and initiation and then monthly/quaterly visits, results from repeat HIV testing, side effects, adverse outcomes and reported adherence	- Within the Sisters Organization: 215 to 315 initations per month of PrEP in 2020 before COVID-19 in the country, in May 2020 increased monthly until 1360 initations in Sept 2020.<br>- From Oct-Dec 2020, there was a national shortage due to delayed shipments<br>- Nationally 746 PrEP initation between jan-march 2020, prior to COVID and then 1161 April to June 2020 with intense restrictions. - After restrictions July to Sept 2020 there were 3084<br>- The number went down to 1548 in Oct-Dec because of a shortage in medication.<br>-Overall, adaptions made facilitated rapid recovery from COVID-19 with peer-led community based delivery. Greater risk perception with sexual based violence and may have led to the openness of using PrEP<br>- Study reported greater gender based violence<br>- The study used community based PrEP services (WhatsAp broadcast, community advocates, Adverse event reporting through WhatsApp and phone and phone medication adherece counselling.<br>-The community advocates were key in identifying high risk FSW and sending them to community clinics
Mbithi et al.	2021	Assessing the real-time impact of covid-19 on tb and hiv services: The experience and response from selected health facilities in nairobi, kenya	Quantitative study	Kenya, Nairobi	Pre COVID March 2019 to Feb 2020 and then COVID March 2020 to Feb 2021	Clinical data from specfic sites	All individuals >18 years old who visited health facilities	During COVID-19 assessment	Pre-COVID-19 pandemic (March 2019- Feb 2020)	HIV testing, referred to ART, diagnosed positive for HIV	- 50.2% decrease in females testing for HIV pre to during COVID. Males and females decrease about the same.<br>-Discussion talks about how women could be particularly affected because difficult to move during lockdown and heightened sense of fear for the family about accessing health faclities.<br>- 1.7% increase in people refered to ART (higher HIV positivity)<br>-HIV testing started to improve in the second six months because community outreach services were implemted.<br>-Did not mention retention on ART
Moyo et al.	2022	Utilisation of HIV services by female sex workers in Zimbabwe during the COVID-19 pandemic: a descriptive phenomenological study.	Phenomenological study/ qualitative study	Zimbabwe, Bulawayo Metropolitian province	Dec 2020 to March 2021	Interviews	Female sex workers	During COVID-19 pandemic	Pre-COVID-19 pandemic (March 2019- Feb 2020)	Accessing HIV services	-Limited access to HIV services<br>- Theme 1) Travelling during COVID-19 with the police present required authorisation letters. In the process of getting the letter HIV status would need to be revealed or empty pill bottles need to be shown or call a clinic nurse. This impacted patient privacy.<br>-Theme 2) during COVID-19 people had trouble getting money working so struggled financially to get food and patients would not be able to take meds on empty stomach/ pay for transport<br>- Theme 3) Decreased health care quality - there was frustration having to wait outside the clinic for COVID-19 screning services (would look bad to clients, doctors would disclose HIV status unintentionlly), poor screening services for viral load/cervical screening, decreased time with service provider so no time to ask questions/get counselling<br>- during COVID-19 there is a need to maintain privacy and confidentiality with HIV care
Mpofu et al.	2021	Distribution of antiretroviral therapy through private pharmacies and postal courier services during COVID-19 in Botswana: acceptability and reach of two out-of-facility individual differentiated service delivery models.	Mixed methods	Botswana, Gaborone	Aug 2020 to Jan 2021	Survey and Interviews	People living with HIV	During COVID-19	N/A	ART use, changed distribution of medication to pharmacies and home ART delivery	- 27/184 females enrolled refused home delivery<br>-87.2% acceptability of home delivery among females (higher than men)<br>- general declining of home delivery because wanted facility pickup, no stable delivery address and workplace constraints<br>- ART home delivery and private pharmacy distribution can reduce the burden on high-volume facilities during COVID

First Author	Year of Publication	Article Title	Study Design	Study Location	Year(s)	**Data Sources** (e.g., pt interviews, questionnaires)	Participants	**Intervention** (e.g., during COVID, post-COVID?)	**Comparator** (pre-COVID?)	**Outcome(s) Measured** (e.g., ART use, clinic visits, # tests)	Relevant Results
Mutyambizi et al.	2021	"Effect of COVID-19 on HIV, tuberculosis, and prevention of mother-to-child transmission of HIV indicators in Mopani district, South Africa"	Quantitative - Descriptive Analysis	South Africa	January, 2020 - March, 2020 and April, 2020 - June 2020	Data from District Health Information System	Pregnant women	During the COVID-19 pandemic	Pre-COVID-19 pandemic	% change in antenatal care visits<br>% change in total HIV-positive pregnant women<br>% change in total HIV-positive pregnant women starting ART<br><br>PMTCT indicators (antenatal visits, total pregnant HIV-positive women identified, # of HIV-positive women started on ART)	- No statistically significant changes for total antenatal care visits and total pregnant HIV-positive women identified from April -December 2019 VS April - December 2020<br>- Modest decrease in PMTCT indicators comparing Jan-March 2020 VS April-June 2020<br>- Total ANC visits somewhat improved in May-Oct 2020, then decreased in November<br>- Statistically significant decline of 24 HIV-positive pregnant women starting ART at the end of the first wave, followed by statistically significant declines in the monthly trend in total ANC visits.<br>**Discussion:**<br>- PMTCT program NOT affected by the lockdown, as it's often provided to people visiting health facilities for other reasons. Antental care visits remained relatively steady during study period, BUT there was a marked increase in May 2020. Increase may be due to pregnant women returning to their homes in rural areas at the end of alert level 5 lockdown.
Nalubega et al.	2021	COVID-19 may exacerbate the clinical, structural and psychological barriers to retention in care among women living with HIV in rural and peri-urban settings in Uganda.	Qualitative study	Uganda, Kampala and Wakiso district	June to July 2020	Interviews	Women who initated ART during pregnancy and some women that were disengaged	During COVID-19-19 pandemic	N/A	Barriers and facilitators for re-enegagement in care among women previously in the B+ (pregnant/breastfeeding women ART program), how these factors impacted by COVID-19	1) Structural barriers- physical, organizational operational factors impacting access to care: transport difficulties, relocation<br>2) Clinical Barriers - fear og contracting COVID-19 at health facility, taking medication on empty stomach<br>3) Psychosocial barriers- HIV stigma, need safe space to take meds<br>Reported Facilitators 1) accessible services (home treatment) 2) supportive systems andh ealth education 3) valuing health 4) disclosing HIV status - resulted in better treatment adherence<br>General Barriers: 1) transport fees 2) clinical barriers including unfriendliness of healthcare staff, or fear of side effects described 3) psychosocial barriers: HIV stigma impacting family
Nyashanu et al.	2021	Exploring the challenges of [illegible]	Exploratory [illegible]	Harare, [illegible]	(Lockdown) 2019 [illegible]	Interview	Women taking ART's	During COVID-19 lockdown	N/A	COVID-19 lockdown on women [illegible]	Transport problems, confusing COVID-19 restrictions, abuse by police, soldiers at roadblocks, a shortage of medication, lack of health check-up routines, [illegible]
Palattiyil et al.	2022	Access to HIV/AIDS or TB care [illegible]	Qualitative Study	Kampala, Uganda	August to [illegible]	Computer Assisted [illegible]	Women refugees recieving [illegible]	Post-COVID-19 pandemic	N/A	HIV/AIDS access to services	Inability to access medication for other comorbidities associated with TB or HIV/AIDS, societal and economical impacts related to retrieval of care: delivery of drugs [illegible]
Rose et al.	2023	"Patient and stakeholder perspectives on impacts of the COVID-19 pandemic on HIV and mental health care delivery in South Africa"	Qualitative	South Africa	No information.	Semi-structured interviews	People living with HIV and healthcare providers	During the COVID-19 pandemic	N/A	- Barriers to HIV care	- Increased structural barriers to obtaining medication ("You know, you end up waiting there for quite some time... (but) I still get my pills. I'm getting all of my pills." - female, patient).<br>- Lack of private & confidential access to providers impacted quality of care. Reduced privacy during care encouters that was brought about by the pandemic (because of social distancing measures at health centers). "Now we're standing there, and they're shouting our names and giving you a pill in front of everyone. There's no privacy at the same time." - female patient.
Siedner et al.	2020	Access to primary healthcare during lockdown measures for COVID-19 in rural South Africa: a longitudinal cohort study	Quantitative Study	Northern KwaZulu-Natal, South Africa	(Lockdown) 2019 to 2020	Suveillane system	Clinical population	During COVID-19 Lockdown	Pre-COVID-19 pandemic	Number of clinic visits, type of clinic (child health/antenatal care, postanatel and family planning/HIV services)	Females were likely to visit ambulatory clinics for HIV services 47.6% druing January 2020 to april 30, 2020, HIV Services increased by 20% after start of the lockdown (from 37.7 to 46.1%)
Silhol et al.	2021	Assessing the potential impact of [illegible]	Quantitative	Yaounde, [illegible]	March 2019 to [illegible]	Clinical Data	Female Sex Workers	Post COVID-19 pandemic	Pre-COVID-19 pandemic	ART services and condom provision, [illegible]	50% reduction in condom use in partnership over 6 months - increase 1-year HIV incidence, among FSW by 42%, COVID-19 related changes in condom use may [illegible]
Skovdal et al.	2023	Condoms are hard to get by': access to HIV prevention methods during lockdown of COVID-19 epidemic in eastern Zimbabwe	Qualitative and exploratory study	Zimbabwe	March 2021 to July 2021	Telephone interviews, group discussions, and photography	Adolescent girls, young women, men	During COVID-19 pandemic	Pre-COVID-19 Pandemic	PrEP services	Access to PrEP was limited because movement was limited, travel permits were needed even to go from the hopital and even when people reached hospital, resources were limied so access to PrEP was limited. Pople were turned away at hospitals and told to adhere to lockdown. Village health workers were giving out PrEP but people were scared to get help out of fear of contracting COVID-19.
Thekkur et al.	2021	Assesing the impact of COVID-19 on TB and HIV programme services in selecte Health Facilities in Lilognew, Malawi:Operational Research in Real Time	Quantitative Study	Lilongwe, Malawi	March 2019 to Feb 2020	Clinical Data	All persons tested for HIV between March 2019 and February 202	During COVID-19 pandemic	Pre-COVID-19 pandemic	Monthly surveillance of TB and HIV treatment and testings.	Pre-COVID-19 pandemic - 147,161 tested for HIV (females) - COVID - 92,209 tested for HIV
Thekkur et al.	2021	Operational research to assess the real-time impact of COVID-19 on tb and hiv services: the experience and response from health facilities in Harare, Zimbabwe	Quantitative Study	Harare, Zimbabwe	March 2020 to Feb 2021	Clinical Data	Patients presenting to HIV treatment and people tested for HIV services	During COVID-19 pandemic	Pre-COVID-19 pandemic	Number of HIV tests administered	62.8% decrease in individuals who got tested for HIV.
Ware et al.	2023	How central Ugandan clinics adapted during COVID-19 Lockdown restrictions to promote continous access to care: A qualitative analysis	Qualitative study	Uganda	June 2018 to December 2021	Individual interviews	Couples	During COVID-19 pandemic	Pre-COVID-19 Pandemic	ART and PrEP initiation, adherence, experience of medicaiton use, PrEP program	Inflexible work days for nursing stff reduced PrEP clinic capacity. The clinics adapted to pre-packaing medicines and having agreed appointments for medicaiton pick-up during COVID-19 and the paperwork was done before-hand. This reducde wait-times. Transport was a barrier for clients, tripled in price or not available, so longer prescription durations were given (multi-month). Clinics created a network to allow clients to receive counseling at multiple locations. Neighburhood delivery was attempted but it was difficult to fine people and there were security concerns. Not all facilitaies had PrEP accessible. any clients were lost ot follow-up because their vllages had no access to PrEP. PrEP was bundled with other medications such as TB meds and ART to address multi-morbidity. HIV self-tesitn kits where widely used to re-confirm HIV negative sttus before refilling PrEP. Many women did not make an income during the lockdown and savings deminished so no lnger could afford PrEP. Financial struggles led people to walk and have their feet swollen, discouraged them from accessing clinics as often.
Wyatt et al.	2022	Influences on PrEP Uptake and Adherence Among South African Women During Periconception and Pregnancy: A Qualitative Analysis.	Qualitative study	South Africa, KwaZulu-Natal	Nov 2017 to July 2021	Interviews	Women during preconception and pregnany phase	Pre and During COVID-19 pandemic	Pre COVID-19 pandemic	Reasons for PrEP uptake or decline, experiences taking PrEP, reasons for missed dose, percieved HIV risk	- Difficulty knowing how to access more PrEP during lockdown<br>- Staying at home made it harder for some, but also made it easier for some<br>- the research study allowed participants to get a referral to a hospital nearby for more medication<br>- many women were in long-distance relationships
